# Using artificial intelligence to identify patients with migraine and associated symptoms and conditions within electronic health records

**DOI:** 10.1186/s12911-023-02190-8

**Published:** 2023-07-14

**Authors:** Daniel Riskin, Roger Cady, Anand Shroff, Nada A. Hindiyeh, Timothy Smith, Steven Kymes

**Affiliations:** 1 Verantos Inc, Menlo Park, CA USA; 2 RK Consults, Ozark, MO USA; 3 Missouri State University, Springfield, MO USA; 4 Axon Therapeutics, San Diego, CA USA; 5 Stanford University School of Medicine, Palo Alto, CA USA; 6 StudyMetrix Research LLC, St. Peters, MO USA; 7 Lundbeck LLC, Deerfield, IL USA

**Keywords:** Migraine, Real-world evidence, Electronic health records, Observational study

## Abstract

**Background:**

Real-world evidence (RWE)—based on information obtained from sources such as electronic health records (EHRs), claims and billing databases, product and disease registries, and personal devices and health applications—is increasingly used to support healthcare decision making. There is variability in the collection of EHR data, which includes “structured data” in predefined fields (e.g., problem list, open claims, medication list, etc.) and “unstructured data” as free text or narrative. Healthcare providers are likely to provide more complete information as free text, but extracting meaning from these fields requires newer technologies and a rigorous methodology to generate higher-quality evidence. Herein, an approach to identify concepts associated with the presence and progression of migraine was developed and validated using the complete patient record in EHR data, including both the structured and unstructured portions.

**Methods:**

“Traditional RWE” approaches (i.e., capture from structured EHR fields and extraction using structured queries) and “Advanced RWE” approaches (i.e., capture from unstructured EHR data and processing by artificial intelligence [AI] technology, including natural language processing and AI-based inference) were evaluated against a manual chart abstraction reference standard for data collected from a tertiary care setting. The primary endpoint was recall; differences were compared using chi square.

**Results:**

Compared with manual chart abstraction, recall for migraine and headache were 66.6% and 29.6%, respectively, for Traditional RWE, and 96.8% and 92.9% for Advanced RWE; differences were statistically significant (absolute differences, 30.2% and 63.3%; *P* < 0.001). Recall of 6 migraine-associated symptoms favored Advanced RWE over Traditional RWE to a greater extent (absolute differences, 71.5–88.8%; *P* < 0.001). The difference between traditional and advanced techniques for recall of migraine medications was less pronounced, approximately 80% for Traditional RWE and ≥ 98% for Advanced RWE (*P* < 0.001).

**Conclusion:**

Unstructured EHR data, processed using AI technologies, provides a more credible approach to enable RWE in migraine than using structured EHR and claims data alone. An algorithm was developed that could be used to further study and validate the use of RWE to support diagnosis and management of patients with migraine.

**Supplementary Information:**

The online version contains supplementary material available at 10.1186/s12911-023-02190-8.

## Background

There is increasing emphasis on the use of real-world data collected as part of routine clinical practice to augment the interpretation of data from randomized clinical trials (RCTs) [1]. Although appropriately designed RCTs are the gold standard of clinical evidence as observed in a patient population, the highly controlled testing environment and study design (i.e., specific selection of patients, rigorous treatment protocols, monitoring, and statistical hierarchies) do not reflect treatment of individual patients cared for in real-world settings [2, 3]. RCTs are an inefficient method to understand patient subgroups and comparative effectiveness of medications. Nonetheless, healthcare providers (HCPs) and payers almost exclusively use RCTs to establish policies on new therapeutic options, as these are considered the best available evidence [3].

The US Congress, under the 21st Century Cures Act, supports the use of real-world evidence (RWE) by the US Food and Drug Administration to support or satisfy post-approval study requirements [4, 5]. RWE is based on information obtained from sources other than clinical studies. These include data from electronic health records (EHRs), claims and billing data, product and disease registries, personal devices (e.g., phones, wearable devices), and health applications (i.e., smartphone apps) [6]. The role and use of RWE is gaining prominence in regulatory, drug development, and healthcare decision-making environments [7]. RWE can complement and validate RCT evidence, extending the understanding of interventions and outcomes based on typical clinical practice patterns in a broader treatment population diagnosed and treated in routine care settings (including those who would be ineligible for RCT due to factors such as age or comorbidities) [2]. This could help policy stakeholders, payers, and HCPs better refine their understanding of the significance and relevance of such evidence in the broader patient population that is likely to receive a given treatment and provide patients an opportunity to voice their experiences and preferences [6, 8, 9]. RWE may support regulatory applications (e.g., new drug indications, post-marketing surveillance) [5], aid payers with coverage and reimbursement decisions, assist physicians in making treatment decisions, and allow patients to be more effective participants in collaborative healthcare decisions [2, 9, 10]. Patients may also benefit because RWE can provide information on treatment patterns across a much broader range of outcomes, including patients who are often excluded from RCTs due to comorbid disease, psychosocial barriers, and ethnicity [2].

Traditional real-world studies rely on insurance claims data with International Classification of Disease (ICD) or Current Procedural Terminology codes providing context or structured EHR data, which leverages the entries made in the predefined fields clinicians use when recording a physician record (e.g., problem list, open claims, medication list, etc.). However, many conditions or procedures require information at a more discrete level of detail than can be gained from these methods [11–14], and there is variability in how routinely collected data are entered in claims or structured EHRs among HCPs [11]. Some HCPs are more comprehensive in their approach than others, with many finding the addition of structured data to be time consuming and inefficient [12, 13]. Additionally, structured data are most commonly used in the billing pathway, which requires limited clinical detail [13].

In contrast, unstructured data from the narrative of the EHRs are used as the medicolegal record and provide HCPs the opportunity to record detail that is essential to patient care, creating an incentive for these data to be more complete; this provides the level of detail necessary for investigators to fully understand the condition or disease progression. However, extracting meaning from the unstructured EHRs for large scale studies requires artificial intelligence (AI) technologies, including natural language processing (NLP) and machine-learned inference, that, if successful, results in the higher-quality evidence for regulators, payers, prescribers, and patients to make their decisions [1]. In this study, data came from the same EHR system which included both structured data and a documentation system for unstructured data. The objective of this study was to develop and validate an approach to identify concepts associated with the presence and progression of migraine using unstructured EHR data. Worldwide, migraine is one of the most prevalent and disabling diseases [15]. The diagnosis of migraine is complex: specific headache history and duration criteria must be met in patients with at least 2 of 4 key migraine-associated characteristics (i.e., unilateral location, pulsating quality, moderate or severe pain intensity, and aggravated by activity) and accompanied by nausea and/or vomiting and/or both photophobia and phonophobia [16]. Furthermore, migraine phenotype requires specific headache and migraine frequency criteria to be met, including a 3-month or longer duration for chronic migraine. Taken together, the prevalence and complex diagnostic criteria make migraine an ideal therapeutic area to assess approaches to obtaining high-quality RWE from EHR data.

## Methods

### Study design

Administration claims and EHR data were sampled from a large US-based integrated health system for the years 2010–2012. Using these data, we compared the accuracy of traditional and advanced RWE approaches for identifying migraine-related concepts relying on chart abstraction with data collected in a tertiary care setting as the reference standard. For purposes of this study, we defined “Traditional RWE” as the use of insurance claims or structured EHR data to identify concepts. Traditional RWE included problem and medication lists and encounter-associated open claims, which were extracted by standard queries by structured query language. “Advanced RWE” was defined as the use of unstructured EHRs to identify relevant concepts and attributes, such as negation or temporality, with AI to process physicians’ notes.

### Technology

To achieve high accuracy, NLP and AI-based inference were used. AI-based technologies were provided by Verantos, Inc. (Menlo Park, CA). NLP supported identification of relevant concepts within the sentence boundary. In situations where insufficient information was available within the sentence or where a concept was suspect, AI-based inference supported identification of patterns within the longitudinal record. Patterns were recognized based on a large corpus of machine-learned healthcare associations as well as inferencing algorithms. For example, in the text “pt with MA”, association with headaches and photophobia may favor disambiguation of “MA” to migraine with aura whereas association with lung cancer and tumor may favor mass. As another example, if a new template includes “-headache, +nausea”, inference may determine that headache is not asserted due to lack of support elsewhere in the encounter.

**Reference standard**

Manual annotation of the data set was used to create a reference standard supporting estimate of recall and precision for extracted data in the Traditional RWE and Advanced RWE arms. In the manual annotation, each concept—and all the metadata associated with each concept—was identified and labeled in the data set. For example, an annotator might mark the text “mod HA” as headache experienced = true, current = true, and severity = moderate. The concepts identified were selected based on recommendations from a local panel of academic and industry headache specialists. Concepts that were chosen reflect features likely to be inclusion and exclusion criteria or subgroup analyses within an RWE migraine study.

The data set consisted of 6,032 encounters. Each encounter was reviewed by 2 clinical annotators and was defined as a single visit for a patient with a single physician on a specific date. During the course of the study, the annotation was performed by 7 annotators, each with ≥ 1 year of medical annotation experience spanning approximately 3,000 training encounters. Inter-annotator agreement was measured by Cohen’s κ score; an average κ score ≥ 0.8 was required for this study. To ensure there was no systematic error, annotator pairings were rotated between a pair of annotators. Any disagreement between the annotators was noted and resolved with discussion between the 2 annotators to arrive at a common agreement. In cases of dispute between the 2 annotators, a third annotator served as the tiebreaker. The κ score was calculated prior to resolution of disagreement.

### Filtering

Preselecting the data set was done to increase the likelihood of relevant concepts and allow for the selection of relevant encounters to annotate. Filtering was implemented to ensure that low prevalence conditions would have sufficient occurrence rates to provide adequate power to demonstrate statistically significant differences between study arms (i.e., Traditional vs. Advanced RWE). Concepts included the terms *migraine* and *headache* and *rizatriptan* and *sumatriptan*. Filtering was applied equally to all data to avoid bias.

### Outcomes and comparisons

Because concepts such as headache or migraine are discrete, time-specific events, concepts were tested at the encounter level, meaning that if a patient had migraine at a specific encounter, it would only be counted as correct in that encounter. It is not assumed that the patient will have migraine throughout the longitudinal record. In each encounter, a specific concept can occur multiple times (e.g., “patient describes frequent headaches” and “the headaches have been severe throbbing”). Thus, concept occurrence is the sum of all occurrences of a specific concept (e.g., migraine). Encounter occurrence is the number of encounters that had ≥ 1 occurrence of a concept. Recall was the primary endpoint used to determine the performance of Traditional RWE and Advanced RWE against the manually annotated reference standard (Table 1). Precision was a secondary endpoint, and F1 scores—the weighted harmonic mean (reciprocal of arithmetic mean with equal weight to each data point) of precision and recall—were calculated as 2 × ([precision × recall] / [precision + recall]) (Table 1). As these are proportions, the chi-square test was used to compare differences in accuracy. For Advanced RWE to outperform Traditional RWE, the protocol required an average recall of at least 80% or an absolute recall difference of at least 25% between the 2 approaches.

**Table 1:** Study Outcomes

Outcome	Endpoint	Definition
Recall	Primary	Proportion correctly identified among those that should have been identified
Precision	Secondary	Proportion correctly identified ÷ total identified
F1 Score		2 × ([precision × recall] ÷ [precision + recall])

## Results

**Fig. 1:**
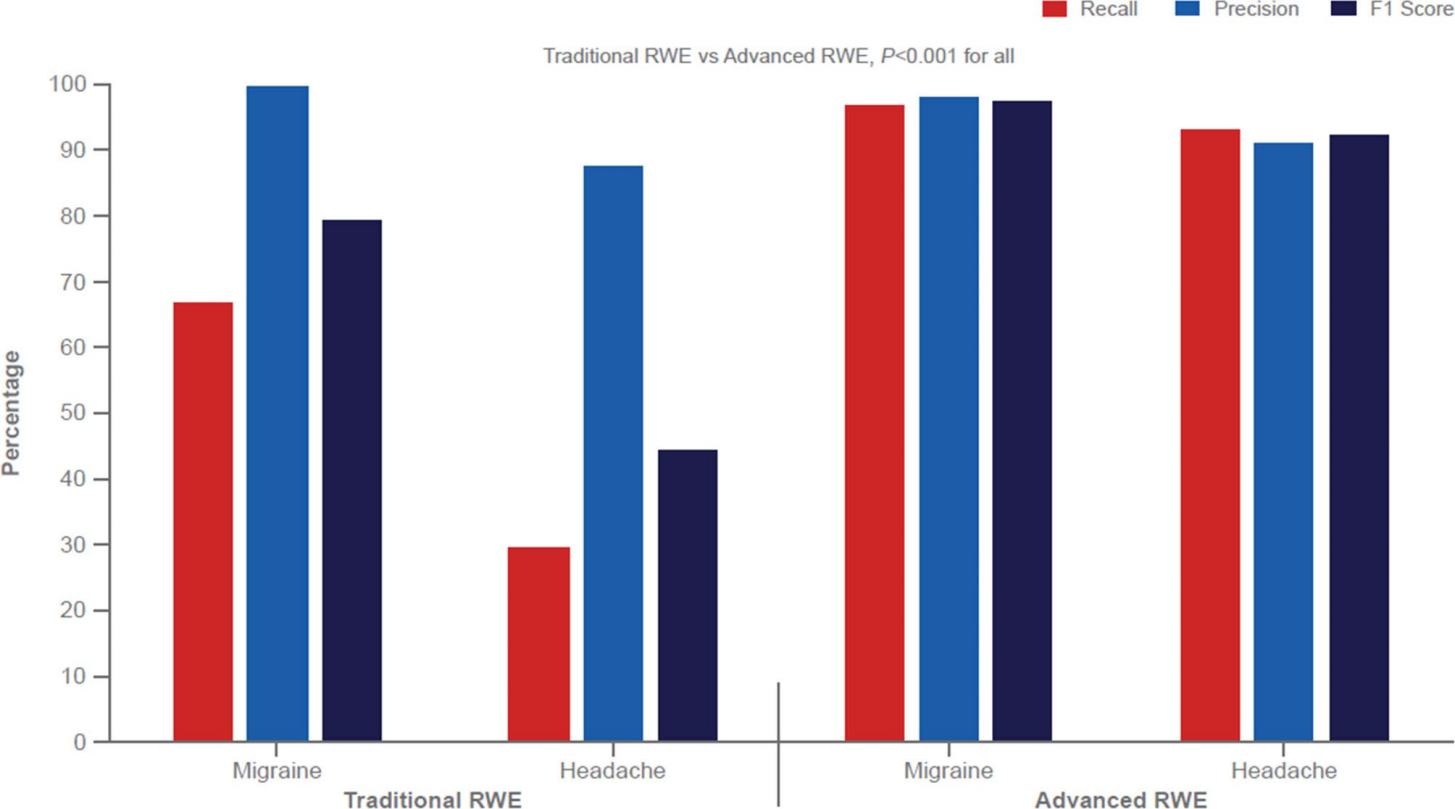
Identification of Migraine and Headache* in Traditional RWE and Advanced RWE RWE = real-world evidence. *Occurrences: migraine, n = 2,642; headache, n = 6,530.

The average interrater reliability Cohen κ score was high (0.9), indicating that manual annotation (i.e., reference standard) was consistent and credible. Concept occurrences for migraine and headache were 2,642 and 6,530, respectively. Recall for migraine and headache were 66.6% and 29.6% for Traditional RWE and 96.8% and 92.9% for Advanced RWE (absolute differences, 30.2% and 63.3% for migraine and headache, respectively; Fig. 1). There were statistically significant differences between Traditional RWE and Advanced RWE for identification of migraine and headache concepts (*P* < 0.001).

**Fig. 2:**
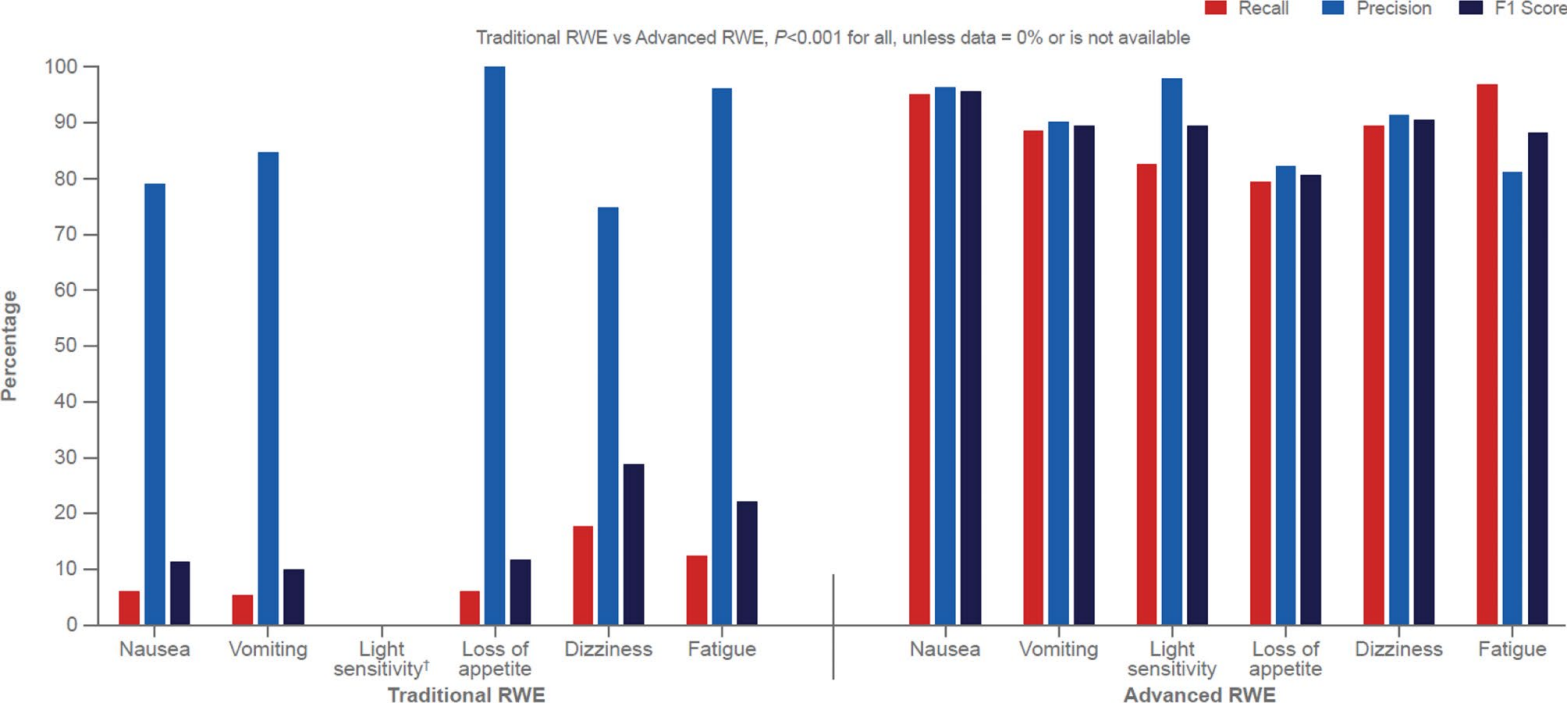
Identification of Migraine-Related Symptoms* in Traditional RWE and Advanced RWE RWE = real-world evidence. *Occurrences: nausea (n = 4,057), vomiting (n = 3,197), light sensitivity (n = 243), loss of appetite (n = 377), dizziness (n = 3,391), and fatigue (n = 4,088). ^†^Recall, precision, and F1 score were 0%, not available, and 0%, respectively.

**Fig. 3:**
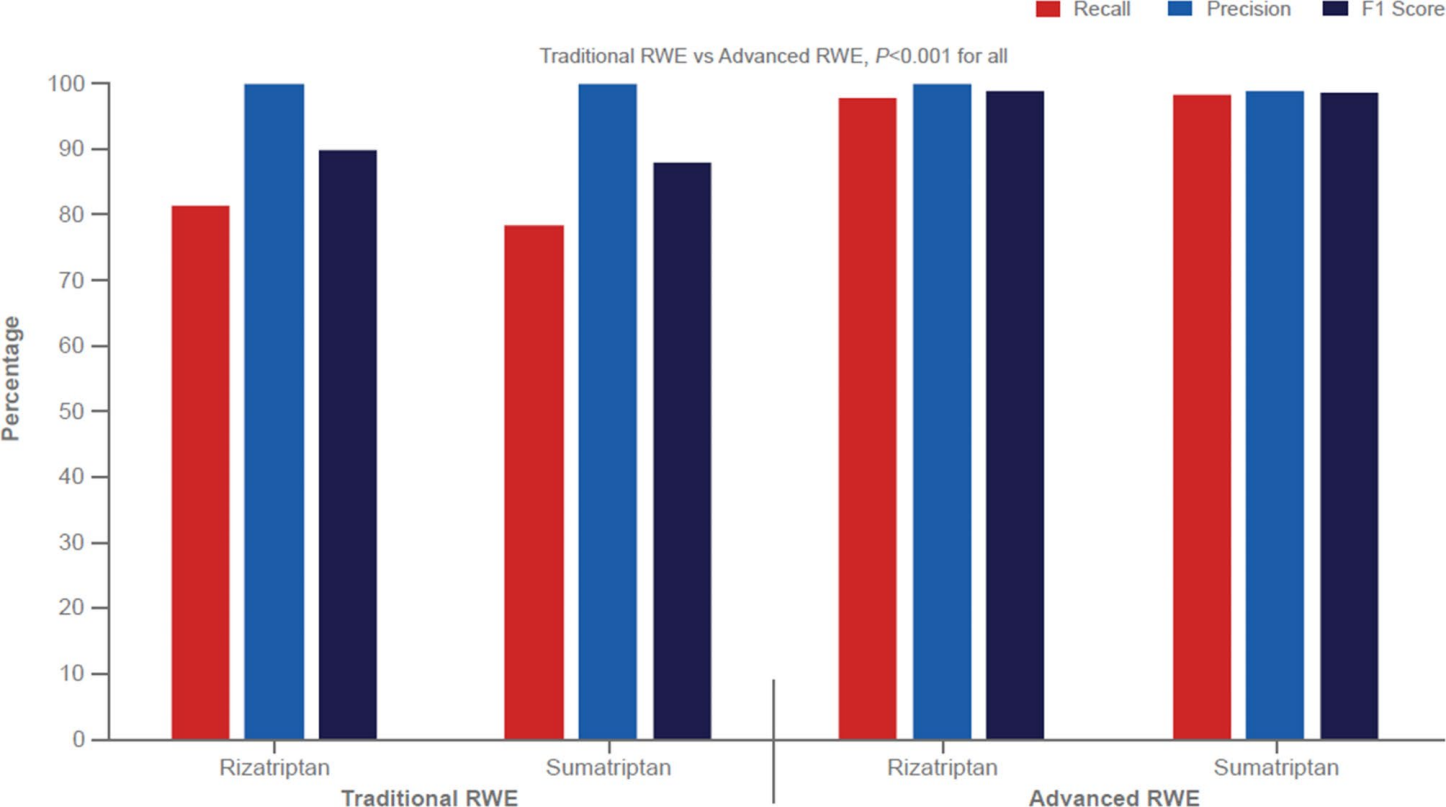
Identification of Migraine Medications* in Traditional RWE and Advanced RWE RWE = real-world evidence. *Occurrences, rizatriptan, n = 102; sumatriptan, n = 510.

For identification of 6 migraine-associated symptoms (243–4,088 concept occurrences), Traditional RWE recall ranged from 0 to 17.9%, whereas Advanced RWE recall ranged from 79.3 to 96.6%, with respective F1 scores of 0.0–28.9% and 80.7–95.6% (Fig. 2). For symptom identification, the absolute differences in recall between Advanced RWE and Traditional RWE ranged from 71.5 to 88.8%; all differences between Traditional RWE and Advanced RWE were statistically significant (*P* < 0.001). For identification of migraine medications (i.e., rizatriptan and sumatriptan; 102 and 510 concept occurrences, respectively), recall was approximately 80% for Traditional RWE and ≥ 98% for Advanced RWE, with F1 scores > 88% for Traditional RWE and ≥ 98% for Advanced RWE (*P* < 0.001; Fig. 3).

## Discussion

In this phenotyping study, we demonstrated that Advanced RWE techniques consistently outperformed Traditional RWE techniques for identification of migraine-related concepts, with a 193% increase in concept recall for advanced relative to traditional approaches. Advanced RWE met the defined study success criteria for identifying patient characteristics (e.g., associated symptoms) in migraine (recall ≥ 80%), whereas Traditional RWE did not (recall range, 0–66.6%). This should not be surprising, as structured data are typically intended for administrative purposes such as filing insurance claims, marketing, or meeting regulatory requirements; as a consequence, critical information necessary for decision making is not likely to be documented in structured data. For instance, there is no ICD-10 code describing the intensity of migraine pain [16]; thus, this omnipresent structured information is not useful for assessing the severity of migraine-related disability or disease progression. Of course, that was not the intent of this administrative and epidemiological tool.

In contrast, symptoms are typically well documented in the EHR narrative, as that is where physicians document impressions to support decisions regarding patient management and prescribing. However, as demonstrated here, these data require use of sophisticated tools, such as AI, to unlock meaning that can be employed by decision and policy makers in regulatory agencies and by payers. There are some decisions that do not require these sophisticated tools; for instance, Traditional RWE was sufficient for identifying prescribed medications (Traditional RWE recall, ~ 80%) and may be the preferred approach for evaluation of interventions because of ease of use and lower cost. Nonetheless, the results of the current study provide support for using Advanced RWE techniques to more accurately identify migraine concepts for evaluating outcome of migraine treatment in a real-world setting.

The use of real-world data necessitates a thorough assessment of their quality and relevance when used for evaluating data from clinical care settings [17]. Unlike data derived from RCTs, which are affected by selection bias (e.g., specific inclusion/exclusion criteria) and nonresponse, data collected in EHRs and claims may be more representative of the real-world setting [17]. However, scientific evaluations and validation of EHR and claims data greatly depend on the quality of the input data and the technology used to extract the appropriate data [2].

Despite the prevalence of migraine and the substantial disability due to migraine [15], very few studies have used EHR data to identify patients with migraine and to characterize their diagnosis, symptoms, or disease progression. A retrospective study using an EHR-based algorithm in a cohort of patients with migraine with multiple comorbidities found that patients with migraine had an increased occurrence of multiple comorbidities compared with a control population [18]. Additional studies using RWE in patients with migraine are needed.

Study arms were separated to provide the best visibility into differential performance of Traditional versus Advanced RWE techniques. This is important in the context of an industry which typically uses single-source claims data only. In practice, combination of datasets can provide the clearest picture into the patient journey. This may include EHR structured and unstructured as well as linkage of national pharmacy and medical claims data and death registry. Combination or linkage of datasets to achieve completeness is outside the scope of this manuscript.

The results of our study provide a novel approach that can be used to extract high-quality data in the migraine population. We believe using this new approach will provide real-world data that allow for a more thorough characterization of patients with migraine and progression of migraine and that support more credible evidence than when low-accuracy data are used. High-validity RWE applied to rich data sources provides a pathway for payers to make informed decisions regarding treatment management, for HCPs to improve understanding of subgroups and comparative effectiveness, and for patients to sustain a better quality of life and reduce the burden of migraine.

A possible limitation of this study is that a tertiary care (i.e., highly specialized care) EHR was used, and the generalization to other healthcare settings is unknown. Variability in language may cause differential performance of technology. Differences in patient populations in terms of severity and effects of treatment are unknown. Additionally, there is a possibility of selection bias favoring Traditional RWE because patients who were likely to have migraine were selected per protocol to provide sufficient frequency of the disease. However, the improvement in recall and precision with Advanced RWE over Traditional RWE methods found in this study is not likely to be an artifact of these factors.

## Conclusions

A specific implementation approach for retrospective EHR-based observational studies in migraine was established. Advanced RWE techniques were required to accurately identify patients with migraine and associated symptoms. A visual summary of the methods and findings is shown in Supplemental Fig. 1. Based on the robust findings of this study, a more reliable approach for RWE studies in migraine could be progressed by using advanced RWE approaches. An algorithm was validated that could be used to further study RWE in patients with migraine.

## Electronic supplementary material

Below is the link to the electronic supplementary material.

Supplementary Material 1

## Data Availability

The datasets generated and/or analyzed during the current study are not publicly available. Lundbeck LLC is committed to responsible sharing of data in a manner that is consistent with safeguarding the privacy of patients, respecting the integrity of national regulatory systems, and protecting the intellectual property of the sponsor. The protection of intellectual property ensures continued research and innovation in the pharmaceutical industry. Deidentified data are available to those whose request has been reviewed and approved through an application submitted to https://www.lundbeck.com/global/our-science/clinical-data-sharing.

## Abbreviations

AI: artificial intelligence
EHR: electronic health record
HCP: healthcare provider
ICD: International Classification of Disease
NLP: natural language processing
RCT: randomized clinical trial
RWE: real-world evidence
