# Supplementary material for: Using artificial intelligence to identify patients with migraine and associated symptoms and conditions within electronic health records
Source: BMC Med Inform Decis Mak. 2023 Jul 14;23:121. doi: 10.1186/s12911-023-02190-8 (PMC10349448; doi:10.1186/s12911-023-02190-8)
Supplement: Supplementary file 1 — Supplementary Material 1 [file 12911_2023_2190_MOESM1_ESM.pdf]

Supplemental Figure 1. Advanced RWE versus Traditional RWE with Unstructured EHR Migraine and Headache Data

Objective: To use unstructured data from electronic health-records (EHR) to develop and validate an approach to identify concepts associated with the presence and progression of migraine in real-world settings.

Conclusion: An algorithm based on unstructured data from EHRs is almost as accurate as manual chart abstraction and better than traditional real-world evidence (RWE) in identifying patients with migraine.

Methods: Algorithms based on traditional and advanced RWE were developed and ability to detect migraine was compared

Data set: EHRs capturing 6032 encounters in a tertiary care setting

Structured EHR data	Unstructured EHR data
Standard query	Artificial intelligence
**Traditional RWE**	**Advanced RWE**

Compared **migraine outcomes** versus manual chart abstraction (2 abstractors)

Findings: Manual abstraction was consistent and reliable. Compared with manual abstraction, recall for migraine and headache and migraine-associated symptoms was significantly higher for advanced RWE than for traditional RWE

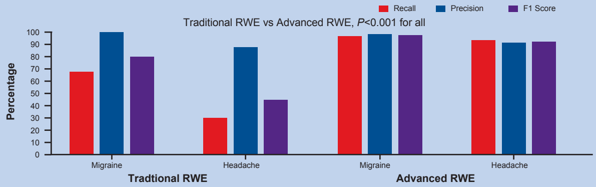

Enhanced recall, precision and F1 score was reported for advanced RWE algorithm compared with the traditional RWE algorithm

EHR, electronic health record; RWE, real-world evidence.
